# Hip resurfacing as an outpatient procedure: a comparison of overall cost and review of safety

**DOI:** 10.1007/s12306-020-00637-z

**Published:** 2020-01-29

**Authors:** M. D. Gaillard-Campbell, C. Fowble, L. Webb, T. P. Gross

**Affiliations:** Midlands Orthopaedics and Neurosurgery, 1910 Blanding Street, Columbia, SC 29201 USA

**Keywords:** Total hip arthroplasty, Outpatient, Hip resurfacing, Minimally invasive, Hip replacement

## Abstract

**Abstract:**

Recent advancements in arthroplasty surgical techniques and perioperative protocols have reduced the duration of hospitalization and length of recovery, allowing surgeons to perform joint replacement as an outpatient procedure. This study aims to evaluate the cost-effectiveness and safety of outpatient hip resurfacing. Two experienced surgeons performed 485 resurfacing surgeries. We retrospectively compared clinical outcomes and patient satisfaction with published outpatient total hip results. Furthermore, we compared average insurance reimbursement with that of local inpatient hip replacement. No major complications occurred within 6 weeks. Of the 39 patients with previous inpatient experience, 37 (95%) believed their outpatient experience was superior. The average reimbursement for hip arthroplasty at local hospitals was $50,000, while the average payment for outpatient resurfacing at our surgery center was $26,000. We conclude that outpatient hip resurfacing can be accomplished safely, with high patient satisfaction, and at a tremendous financial savings to the insurer/patient.

**Level of evidence:**

III

**Electronic supplementary material:**

The online version of this article (10.1007/s12306-020-00637-z) contains supplementary material, which is available to authorized users.

## Introduction

Joint replacement has entered an era of greater minimally invasive alternatives, which provide patients with options to decrease recovery time and financial expense. The three major cost drivers of joint replacement surgery include the price of the implant, extraneous hospital fees, and postoperative rehabilitation expenses [1]. Although minimizing length of stay would reduce these costs, several factors often necessitate overnight hospitalization for arthroplasty patients, including pain control, blood loss, and monitoring of comorbidities. However, with recent advancements in analgesia technology, implant design, surgical technique, and postoperative management [2], many practices now offer outpatient surgery as an alternative to decrease healthcare costs and expedite patient recovery [1].

Although several centers have shown that total hip arthroplasty (THA) can be performed safely and cost effectively as an outpatient procedure [1, 3–5], there have been no reports on outpatient hip resurfacing arthroplasty (HRA), to our knowledge. Many factors could be responsible for the lack of published outpatient outcomes on HRA, including that resurfacing often requires a longer learning curve [6–8], sometimes requires longer operative times, and results in greater rates of failure when performed by less-experienced resurfacing surgeons [9].

However, we have not experienced these issues at our practice, except for overcoming the initial learning curve many years ago. Our practice focuses on HRA in younger and healthier patients whom might be ideal candidates for outpatient surgery; therefore, we contracted with one major insurer to begin a pilot program offering outpatient joint replacement at our surgeon-owned center. This report focuses on the first consecutive series of HRA done under this program.

The purpose of this study is threefold: (1) We evaluate outpatient cost-effectiveness by comparing average insurance reimbursement for outpatient HRA at our facility with the mean insurance reimbursement for inpatient hip replacement at local hospitals. (2) Next, we assess the safety of outpatient HRA by comparing our early clinical outcomes with published results on outpatient THA. (3) Lastly, we compare our satisfaction scores with published results to assess overall patient gratification and approval of their outpatient experience. We hypothesize that outpatient HRA at our facility generates comparable, and oftentimes superior, clinical outcomes than outpatient THA; further, we hypothesize that this alternative increases patient satisfaction while decreasing the cost to patients and/or insurers as compared to inpatient hip arthroplasty.

## Materials and method

Between May 2012 and March 2018, two experienced surgeons performed 485 hip resurfacings as an outpatient procedure in a privately owned surgical center in Columbia, SC. This consecutive series of initial outpatient HRA procedures constitutes 40% of total HRAs performed by these surgeons during this time. Only local patients were initially enrolled, but with further experience and refinement of surgical technique and perioperative protocols, we began offering the procedure to qualifying out-of-state patients. Inclusion criteria comprised patients under 65 years old (excluding one healthy 71 years old and one 42 years old with a BMI of 46 but otherwise healthy), without major comorbidities (such as cardiovascular or pulmonary disease), and without a history of narcotic dependence. Table 1 lists patient demographic information and diagnoses. The study group comprised 342 men (71%) and 143 (29%) women.

**Table 1:** Demographics

Variables	Number	Percentage
# of cases	485	–
In women	143	29.4%
In men	342	70.5%
*Diagnosis*		
Osteoarthritis	320	66.0%
Dysplasia	43	8.9%
AVN	37	7.6%
Trauma	4	0.8%
Other	81	16.7%
	*Average*	*Range*
Age at surgery (years)	53 ± 7	32–71
BMI	28 ± 5	18–46

Table 2 presents a summary of surgical information. All operations were performed through a posterior, minimally invasive, vascular-sparing surgical approach, with an average incision length of 4 ± 0.5 inches using the uncemented Biomet Magnum-ReCap^®^ metal-on-metal hip resurfacing implant system [10]. This device was used in an off-label fashion. All patients were discharged from the ambulatory surgery center before 11:59 p.m. the day of surgery; up to five cases were performed in a day by each surgeon.

**Table 2:** Laboratory results

Variables	Mean	Range
*HBG*		
Preop	14.9 ± 1.6	11.0–20.5
RR	13.5 ± 1.3	9.4–17.3
PoD1	11.9 ± 1.2	9.0–14.9
Na PoD1 (mmol/L)	139 ± 2.8	128–146
K PoD1 (mmol/L)	4.1 ± 0.3	3.4–5.3
CR PoD1 (mg/dL)	0.9 ± 0.2	0.5–2.3

Our outpatient protocol included patient selection criteria, preoperative medical clearance guidelines, minimally invasive surgical techniques, and comprehensive blood management, infection prevention, and pain management programs. Detailed descriptions of each protocol are listed in Supplemental Table 1.

Postoperatively, athletic trainers advised patients on how to walk and climb stairs with crutches, depending on the patient’s weight-bearing protocol. Trainers also encouraged patients to walk as far as they were comfortable, beginning on the first postoperative day and gradually increasing the number of steps taken daily. Table 3 summarizes average distance walked on each postoperative day; this is recorded by a physical therapist on postoperative day 1. Afterward, patients measure with either a wearable device or phone app.

**Table 3:** Average postoperative distance walked

Feet walked postoperative days	Mean	Range
Day 1	296 ± 316	0–3000
Day 2	528 ± 620	25–5280
Day 3	685 ± 761	20–4224
Day 4	911 ± 1394	35–13,200
Day 5	985 ± 1232	20–10,560

We asked patients to return for routine follow-up once within 1–4 days postoperatively and then again at 6 weeks postoperatively. All patients were also followed long term at regular intervals, with the accumulated data maintained in a comprehensive database. However, this report focuses on the results achieved in the first 6 weeks. Patients were asked to record clinical data on a one-page chart and return it at their 6-week visit. At this follow-up appointment, patients were given a 13-question visual analog satisfaction survey [11]. We collected completed satisfaction surveys for 302 patients (66%). Six-week follow-up information was recorded for 304 patients (66% of cases); this information includes data on pain, activity, range of motion, and radiographic implant measurements. Of the 304 patients returning for their 6-week follow-up, 238 patients (78%) returned their one-page data sheet. All patients returned for follow-up by 2 years, but later follow-up results are outside the scope of this paper.

We obtained the average inpatient reimbursement when hip replacement is performed at local hospitals from Blue Cross Blue Shield of South Carolina’s Member Portal [12], which is reported as including all costs from check-in to checkout; the portal does not distinguish pricing between THA and HRA, instead reporting values for general hip replacement only. For this reason, we were unable to directly compare outpatient and inpatient HRA costs. We derived the outpatient reimbursement when HRA is performed at our ambulatory surgery center by aggregating the payments remitted by patients and their insurers for the facility, surgeon, surgeon’s assistant, and anesthesia providers for each HRA procedure to determine a case rate. Blue Cross Blue Shield identifies professional services and procedures with CPT, HCPCS, and ICD codes, from which they generate claims for reimbursement.

A retrospective analysis of our prospective database was performed with IRB approval granted by the IRB Committee at Providence Health in Columbia, SC. Due to the lack of other published studies on outpatient HRA, clinical outcomes were compared to published results for outpatient THA. Student’s t tests for numerical variables and two-sample Z tests for proportions were performed using SAS^®^ (SAS Institute Incorporated, Cary, NC, USA). All tests were performed using a confidence interval of 95%.

## Results

Reports obtained from the Blue Cross Members’ Portal [12] indicate that the average of all costs from check-in to checkout for services rendered in the performance of inpatient hip arthroplasty at our three local hospitals are $39,000, $43,000, and $67,000. The corresponding cost when HRA is performed at our outpatient surgery center is $26,000. This differential represents savings to the patient and/or insurer between $13,000 (33%) and $41,000 (61%). Among our first 485 consecutive outpatient resurfacing cases, there were two emergency room visits and one hospitalization for minor complications. Nine patients (1.9%) required a single liter of intravenous fluid by the visiting nurse after discharge from the surgery center. There were no failures noted in the first 6 weeks postoperatively. There were ten complications (2.0% of cases) not requiring revision. Two patients suffered from acute urinary retention; both resolved spontaneously. Two patients experienced severe constipation, both of which required an ER visit or hospitalization. Another patient experienced a nonsymptomatic cup shift; the implant is now well fixed, and the patient did not suffer any subsequent complications. Two patients experienced a fall on the first postoperative day, one of which was related to persistent symptomatic fascial dehiscence; this was diagnosed with an MRI immediately after the fall and was repaired electively 8 months postoperatively. One patient experienced an anxiety attack within 1 week postoperatively, although it is unknown if this was related to their surgery. Another patient suffered a muscle spasm and was prescribed muscle relaxers. Lastly, one patient experienced abnormal chromium levels (2.4 µg/L) on postoperative day 1 which was noticed on routine laboratory work. The surgeon recommended increased hydration, and the patient’s chromium dropped to 1.3 µg/L by postoperative day 6.

Mean operation time was 88 ± 12 min. Mean blood loss was 173 ± 58 cc. Table 2 lists complete blood count, electrolyte panel, and whole blood metal ion test results. Hemoglobin levels remained above the transfusion trigger for all patients, at an average of 11.9 g/dL on postoperative day 1.

**Fig. 1:**
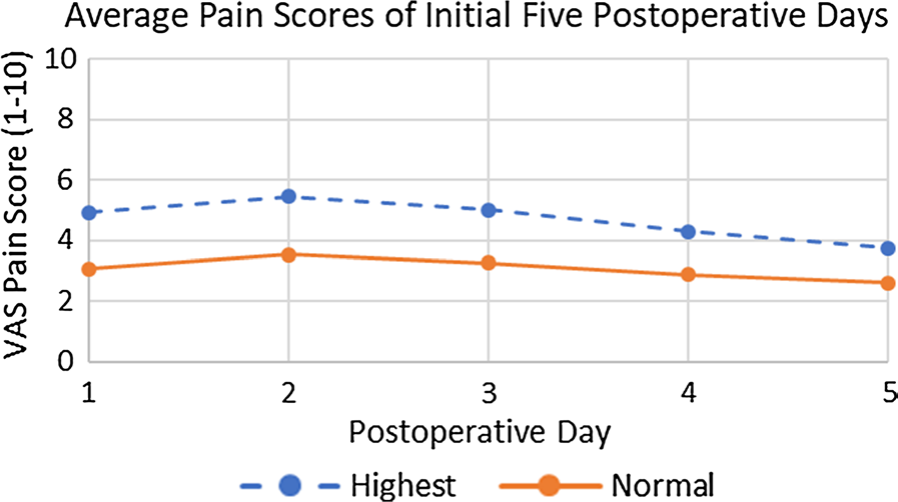
Visual analog scale (VAS) pain scores. Each patient recorded their own subjective VAS pain score for their normal, or “average,” mean pain and their worst, or “highest,” mean pain of the day. The pain scale ranged from 0, or no pain, to 10, or maximum, debilitating pain

**Fig. 2:**
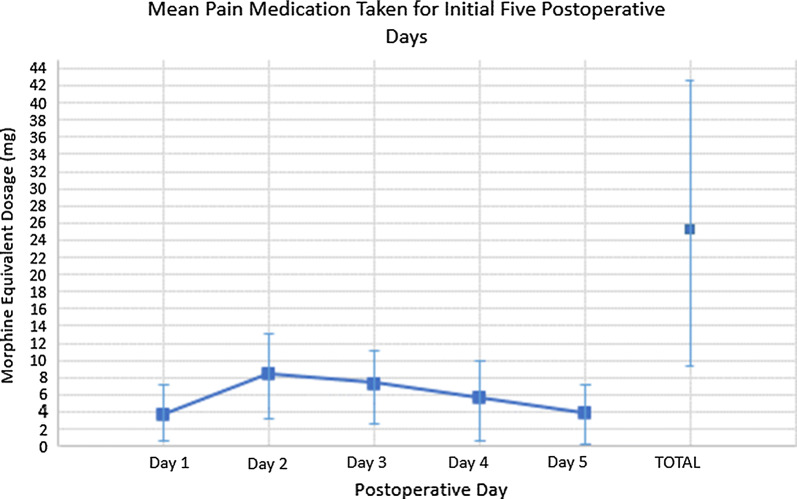
Narcotics utilization. All postoperative narcotics administered were converted to 1 mg morphine equivalents. A mean total of 26 mg morphine equivalents were used during the first 5 days postoperative

On an analog pain scale from 1 to 10, with 1 representing no pain and 10 representing maximum pain, patients reported an average pain score of 3.1 over the first 5 days postoperatively. Figure 1 graphically presents mean pain scores plotted over this time; “highest” and “average” pain levels are shown, which indicate both peak and normal pain levels for each patient, respectively. Patients were given both long- and short-acting narcotics and recorded the amount taken. We converted all narcotics to normalized 1 mg morphine equivalents; Fig. 2 displays these converted values graphically. Mean total morphine equivalents required for the first 5 days postoperatively were 26 ± 17 mg (range 0–69). Patients did not require additional narcotics after the first 5 days. Before incorporating Exparel into our perioperative protocol, seven patients (1.4%) were given a single injection of morphine by the home health nurse on the night of surgery.

**Fig. 3:**
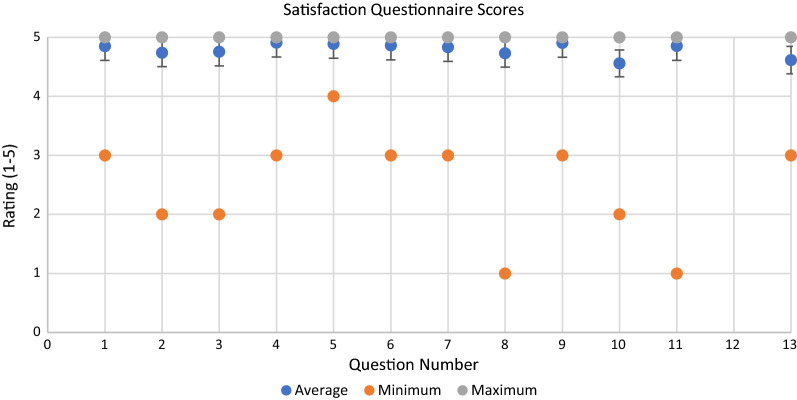
Satisfaction questionnaire. Mean, minimum, and maximum scores for each satisfaction questionnaire item. Item 12 is a yes/no question and is therefore not graphed

We used a satisfaction survey (Fig. 3) to quantitatively summarize overall patient satisfaction (Table 4). On a scale from 1 to 5, with 5 being “strongly agree” or very pleased with that aspect of their experience, each survey item was answered with a mean score between 4.6 and 4.9. Of the 39 patients with previous inpatient surgery experience, 37 (95%), rated their outpatient experience as superior, with the remaining 2 (5%) patients feeling that neither experience was any better or worse than the other.

**Table 4:** Mean satisfaction questionnaire scores

Question #	Mean	Range
1	4.9 ± 0.4	3–5
2	4.7 ± 0.6	2–5
3	4.8 ± 0.5	2–5
4	4.9 ± 0.3	3–5
5	4.9 ± 0.3	4–5
6	4.9 ± 0.4	3–5
7	4.8 ± 0.4	3–5
8	4.7 ± 0.6	1–5
9	4.9 ± 0.3	3–5
10	4.6 ± 0.7	2–5
11	4.9 ± 0.4	1–5
12	39 yes	–
13	4.6 ± 0.6	3–5

## Discussion

Outpatient arthroplasty presents the potential to reduce surgical expense by minimizing postoperative duration of stay. However, there is currently no published literature on outpatient HRA. This study presents the short-term outcomes of our first 485 consecutive cases of outpatient HRA performed by two experienced resurfacing surgeons under a pilot program with one major insurer (Blue Cross Blue Shield of South Carolina). We achieved substantial cost savings for the patient consumer and his/her insurance carrier with no compromise in safety or patient satisfaction. This paper only describes joint surgery policy in the USA.

While traditional cost analyses attempt to itemize the actual cost of supplies, personnel and facility resources for the hospital, our study focuses on the cost to the consumer and his/her insurance carrier represented by the actual payment due to the provider for services rendered per the patient’s insurance plan. Consumers are interested in the actual cost of a service to the hospital to the extent it directly correlates to their out-of-pocket expense; therefore, we define consumer cost as the total dollar amount paid by both the patient and their insurance carrier for the rendered service. Our study provides a comparison of consumer cost of hip arthroplasty between local facilities (Columbia, SC, USA) using data provided by one dominant insurer in our region.

Cost savings at our facility, when compared with local inpatient hip arthroplasty costs, ranged between $13,000 (33%) and $41,000 (61%), with an average savings of $24,000 (48%). Patient satisfaction questionnaire data indicated that overall approval of the outpatient HRA procedure at our center was rated highly. An overwhelming majority of patients with previous inpatient experience (95%) rated their outpatient experience as superior. These data come from our earliest set of outpatient cases; as we grow beyond this initial outpatient learning curve, we expect further improvement in clinical outcomes and patient satisfaction. We believe that with this expectation, and with the current data presented, that extended approval of our outpatient HRA procedure will be granted by a greater number of insurance companies.

We know of no other reports on outpatient HRA. However, there have been several reports on outpatient THA [2, 13–17]. Berger has published numerous articles on his approach [2, 13, 14]; in 2009, his group investigated 150 consecutive THA outpatients. They employed similar inclusion criteria and pain management protocols as we did, and overall, their results were similar. However, they only discharged the first patient of the day as an outpatient, while our surgeons independently performed up to 5 consecutive outpatient cases in a day. They had a significantly larger range of blood loss (100–1000 cc), despite having minimal average loss (286 cc) similar to our cohort (170 cc). Next, they routinely used autologous blood transfusions, while we did not. Further, they used aspirin for thromboprophylaxis, while we employed Xarelto. Perhaps the greatest difference was their procedures which were performed in a large hospital setting, while ours were performed in a freestanding, outpatient surgical center. Berger concluded that “savings to the hospital in length of stay may be outweighed by the additional costs of personnel” [13]. But even at a significantly lower charge than our local hospitals, we profitably performed outpatient HRA. In a separate 2005 cost comparison study [4], Bertin concluded that outpatient THA charge was $2500 less than inpatient THA at the same hospital.

We acknowledge some key limitations to this study. First, we implemented a selection bias, electing only younger, healthy patients without major comorbidities to receive outpatient HRA. The outpatient setting may not be the best alternative for patients with underlying conditions, as these comorbidities often need to be monitored overnight by medical staff. Next, we were unable to compare the results of our cost analysis with national averages, as these values range significantly ($10,000–$75,000) [18]. Third, we limited the scope of this paper to only the first 6 weeks postoperatively, although we have routinely collected information on these patients well beyond this interval. However, the purpose of this study was to evaluate consumer cost and safety of HRA without a hospital stay rather than determining long-term outcomes of the procedure. Lastly, the reproducibility of this study will be difficult without an experienced HRA surgeon, as resurfacing is reported to have a steep learning curve [19–21].

We conclude that in properly selected patients, outpatient hip resurfacing can be accomplished safely, with a high degree of patient satisfaction, and at a significant cost savings to the consumer/insurer. As out-of-pocket medical costs to patient consumers continue to increase, a cost/benefit analysis must be made. Our data indicate that healthy patients who qualify for outpatient surgery may realize substantial savings, a high degree of satisfaction, and an extraordinarily low rate of complications. We intend to gradually expand indications to allow more patients to take advantage of this option. Currently, we are limited in large part by the lack of acceptance of outpatient joint replacement by insurance companies. Based on the evidence presented herein, we encourage insurers to reevaluate their policies and to begin incentivizing patients to take advantage of the highest-quality, lowest-cost surgical options. This study reveals the tremendous healthcare savings that could be generated with wider availability of outpatient hip resurfacing and at no compromise to safety or patient satisfaction.

## Electronic supplementary material

Below is the link to the electronic supplementary material.

Supplementary material 1 (DOCX 17 kb)

## Abbreviations

THA: Total hip arthroplasty
HRA: Hip resurfacing arthroplasty
