# Supplementary material for: Hip resurfacing as an outpatient procedure: a comparison of overall cost and review of safety
Source: Musculoskelet Surg. 2020 Jan 29;105(1):111–6. doi: 10.1007/s12306-020-00637-z (PMC7960592; doi:10.1007/s12306-020-00637-z)
Supplement: Supplementary file 1 — Supplementary material 1 (DOCX 17 kb) [file 12306_2020_637_MOESM1_ESM.docx]

**Supplemental Table 1: Midlands Orthopaedics Outpatient Joint Protocol**

| **1. Patient Selection** | | |
| --- | --- | --- |
| *Requirements* | *Contraindications* | |
| - Blue cross insurance or self-pay - Under 65 years old - Strong family support available | - Major cardiovascular or pulmonary disease - ASA > 2 - Habituated to narcotics - Narcotic intolerance - Severe prostate disease - Psychologically fragile: psychoactive drugs, fibromyalgia, or more than 3 allergies reported - Sleep apnea - BMI > 35 - Requires complex primary surgery - Other major physical disabilities | |
| **2. Pain Management** | | |
| *Preoperative* | *Intraoperative* | *Postoperative* |
| - Celecoxib 200 mg or Meloxicam 15 mg - Scopolamine patch - Oxycodone 20 mg - Ondansetron 4 mg IV - Dexamethasone 8 mg IV | - Spinal anesthetic - Bupivacine local subcutaneous wound injection - Platelet concentrate | - Tapentadol ER 100 mg or Oxycodone 20 mg bid - Celecoxib 200 mg or Meloxicam 15 mg QD - Ice machine - Acetaminophen 1000 mg thrice daily - Oxycodone 5-10 mg every 4 hours as needed - Ondansetron ODT 4 mg as needed for nausea |
| **3. Blood Management** | | |
| *Preoperative* | *Intraoperative* | *Postoperative* |
| - Hemoglobin (Hg) check 1-2 months prior to surgery   - If Hg<15, prescribe iron   - If Hg<13, prescribe iron and retest in 1 month   - After 1 month, if Hg<13, prescribe erythropoietin - Stop aspirin, NSAIDs, and antiplatelet agents for 6 days prior to surgery | - Minimally invasive surgery - Auquamantys tissue sealer - Betadine/epinephrine irrigation - Platelet concentrate with thrombin | - Transfusion trigger Hg<7 |
| **4. Infection Prevention** | | |
| *Preoperative* | *Intraoperative* | *Postoperative* |
| - Screen for active infection/ Medical clearance gained if…   - Hg AIC<7   - Prealbumin>190 mg/L - Hibiclens shower the night before surgery and the AM of surgery   - Surgical area shaved with electric razor and painted with Chlorhexidine - Mupirocin applied to nares 3 days prior to surgery and in the preoperative holding area - Ceftriaxone 2 g IV prior to incision, or Clindamycin if allergic to penicillin | - Clean air operative room (>25 air changes/hour with HEPA filter) - Duraprep, plastic betadine impregnated adhesive drapes - Minimize bleeding according to the Blood Management Protocol - 1 g Vancomycin powder on implants - Platelet concentrate - Jet lavage with 3 L dilute betadine and epinephrine - Tight multilayer closure with Quill absorbable suture | - Silver impregnated, waterproof dressing for 7 days - Bactroban daily to wound thereafter for several weeks - Oral antibiotic coverage for significant wound drainage |
| **5. General Postoperative Protocol** | | |
| - Family is allowed to sit with patient immediately on arrival from operating room. - 1-liter Ringers lactate intravenous bolus upon arrival in recovery area. - Foley removed when feet begin moving. - Narcotics titrated as spinal wears off. - Athletic trainer teaches simple leg exercises and crutch walking, and stair climbing according to weight bearing protocol assigned. - Knee immobilizer if quad is not active. - Flomax prescribed for 1-week before and after surgery for men over 50. - Bladder scan after first void to assure adequate bladder emptying. - Patient is fed and encouraged to drink copious Gatorade. - Supine and standing pelvis x-rays are obtained. - Written instruction reviewed with patient and family. - Family instructed on how to fill out data sheet. - All prescription medications directly given to patient. - Patient is discharged to home or hotel. - Home health nurse visits evening of surgery and the following morning. - Out of area patients are visited in the hotel by the surgeon prior to return trip home. - Local patients are seen in the office 1-week after surgery. | | |
